# Association between serum vitamin D and chronic rhinosinusitis: a meta-analysis

**DOI:** 10.1016/j.bjorl.2019.08.007

**Published:** 2019-10-03

**Authors:** Bo Li, Miaowei Wang, Lingyun Zhou, Qiao Wen, Jian Zou

**Affiliations:** a Sichuan University, West China Hospital, Department of Otorhinolaryngology, Head & Neck Surgery, Sichuan, China; b Sichuan University, West China Hospital, Center of Rehabilitation, Sichuan, China; c Sichuan University, West China Hospital, Center of Infectious Diseases, Sichuan, China

**Keywords:** Sinusitis, Nasal polyp, Vitamin D, 25-Hydroxyvitamin D, Meta-analysis

## Abstract

**Introduction:**

Chronic rhinosinusitis is a highly heterogeneous chronic inflammation of the upper respiratory tract caused by immune dysfunction in human beings. However, the underlying etiology of this disease has not yet been well established. Several trials have revealed that serum vitamin D level abnormality might play a role in the pathophysiology of chronic rhinosinusitis.

**Objective:**

The aim of present study was to perform a meta-analysis of studies to compare the serum vitamin D levels between patients with chronic rhinosinusitis and healthy controls and to evaluate the potential associations of serum vitamin D level with the occurrence of chronic rhinosinusitis.

**Methods:**

Following the PRISMA guidelines, relevant databases incorporating Pubmed, Web of Science, Embase and Cochrane Library were searched from inception to December 1, 2018. Funnel plot symmetry and Egger’ test were used to detect publication biases. The outcomes were presented as Weighted Mean Difference combined with 95% Confidence Intervals to estimate the difference of serum vitamin D levels between patients with chronic rhinosinusitis and controls. Higgins I^2^ value was used to test for heterogeneity between the included studies.

**Results:**

Out of 176 studies, only eight studies involving 337 chronic rhinosinusitis patients and 179 healthy controls met the criteria and were included in the meta-analysis. In a pooled analysis of the studies, chronic rhinosinusitis patients showed decreased serum vitamin D status (WMD = −7.80, 95% CI −13.28 to −2.31, *p* = 0.000). Subgroups analyses based on study location (USA vs. Non-USA), types of biomarkers (25(OH)D3 vs. 25(OH)D) and the study design methods (retrospective vs. prospective) did not reveal heterogeneity. However, phenotypes of chronic rhinosinusitis, with versus without polyposis might account for some degree of heterogeneity. Meanwhile, a lower serum vitamin D level was observed in chronic rhinosinusitis with nasal polyps patients.

**Conclusion:**

Our findings indicate that serum vitamin D level might be associated with patients with chronic rhinosinusitis as we detected a significant association between lower serum vitamin D status and chronic rhinosinusitis, especially in chronic rhinosinusitis with nasal polyps patients. However, further comprehensive studies are warranted in order to draw firm conclusions.

## Introduction

Chronic rhinosinusitis (CRS) is a disease of the nasal and paranasal mucosa characterized by the persistent inflammation with distinctive inflammatory cells. Epidemiologic studies have revealed wide variation in the prevalence of CRS among regions globally.^1^ According to the data of 2012 European Position Paper on Rhinosinusitis and Nasal Polyps (EPOS2012), the overall prevalence of CRS is 5%–15% in Western populations. In addition, CRS is ranked as one of the top 10 costly healthy conditions to US employers, overcoming asthma costs.^1^, ^2^ Thus, we believe that CRS is still an undervalued disease and represents a large socioeconomic burden.

The specific pathogenesis of CRS isn’t totally clear. In the past, CRS was considered to be a chronic suppurative inflammation caused by bacterial infection. At present, more and more studies have demonstrated that CRS is a highly heterogeneous chronic inflammation of the upper respiratory tract caused by immune dysfunction in human beings.^3^ Based on the radiologic and endoscopic findings, CRS could be divided into two distinct clinical phenotypes: CRS with nasal polyps (CRSwNP) and CRS without nasal polyps (CRSsNP). The immunologic mechanism of these two phenotypes is different. Briefly, CRSwNP is characterized as an end product of Th2 cell skewing, mediated by IL-4, IL-5, IL-13. In contrast, CRSsNP is typically considered a result of a Th1 inflammation via, with dominant production of IFN-γ.^1^

It is generally considered that vitamin D could maintain the healthy balance of calcium and phosphorus playing an important role in the bone metabolism.^4^ Increasing number of studies have revealed that vitamin D has a wide range of biological functions, not only in the calcium and phosphorus metabolism, but also in hormone secretion, cell proliferation and differentiation. As an immune-modulatory steroid hormone, Vitamin D3 (VD3) directly regulates a variety of cell types, including monocytes, macrophages, epithelial cells, dendritic cells and T-cells. Through blocking monocyte to DC differentiation and maturation and thus diminishing DCs stimulation of T cell Th1/Th2 differentiation, vitamin D influences the process of immune response.^5^, ^6^ Although the exact mechanisms remain unclear, recent evidences support that vitamin D might play an important role in the pathophysiology of CRS. Serum 25-Hydroxyvitamin D (25(OH)D) levels are considered the chief circulation forms of vitamin D and are representative of body vitamin D status. Besides, some authors point out that VD3 seemed more appropriate than vitamin D2 to sustain adequate levels of 25(OH)D and that vitamin D deficiency was associated with CRSwNP.^7^, ^8^, ^9^

To the best of our knowledge, no systematic evaluation and meta-analysis have been conducted on the relationship between serum vitamin D levels and CRS or healthy controls. Up to now, this is the first meta-analysis to investigate the possible associations between serum vitamin D status and CRS.

## Methods

### Search strategy

Our meta-analysis was performed according to the recommendations of the Preferred Reporting Items for Systematic Reviews and Meta-Analyses (PRISMA).^10^ Multiple databases and related search engines were used in this study. PubMed, Embase, Cochrane Library and Web of Science databases up to December 1, 2018 were comprehensive searched for identify eligible articles using the terms “vitamin D [MeSh Terms]” OR “25-hydroxyvitamin D[Title/Abstract]” OR “25(OH) vitamin D [Title/Abstract]” OR “25(OH)D[Title/Abstract]” OR “cholecalciferol[Title/Abstract]” OR “calcitriol[Title/Abstract]” OR “ergocalciferols[Title/Abstract]” combined with “chronic rhinosinusitis[Title/Abstract]” OR “chronic sinusitis[Title/Abstract]” OR “CRS[Title/Abstract]”. References of articles and reviews were also searched manually for additional potentially eligible studies. Only the most recent studies with sufficient data reported were kept when the data showed to be duplicated in different studies.

### Inclusion and exclusion criteria

Studies were included in the meta-analysis if they met the requirements below: (1) Studies reporting comparison of serum vitamin D levels between CRS and controls; (2) Articles had a clear definition of cases and controls; (3) Vitamin D measured using a standard method and expressed as, or could be converted to, one international unit (ng/mL);^11^ (4) Articles published in English.

The exclusion criteria studies were: (1) Patients with other risk factors, including asthma, allergic rhinitis, fungal infection, nasal tumor and so on; (2) Animal studies, abstracts, letters, editorials, reviews, expert opinions; (3) Articles did not support sufficient data; (4) The full text was not available in English. The final decision was obtained by consensus.

### Study selection and data extraction

Two independent researchers reviewed all candidate articles for inclusion or exclusion. Any conflicts in the data extraction or quality assessment, were resolved by a third reviewer. The data were extracted and recorded in a data extraction form. The following data were collected: first author, publication year, region or country, study design, number of participants, basic characteristics of the individuals, mean of vitamin D level and their standard deviations, vitamin D cutoff value. If necessary, we would contact the corresponding author of the article to obtain unpublished data. The study would be excluded after two failed attempts.

### Quality assessment

The Newcastle-Ottawa Quality Assessment Scale (NOS), which consists of three parts: selection of study groups (4 scores), comparability of groups (2 scores), and outcome assessment (3 scores), was used to assess the quality of the enrolled studies.^12^ Studies quality was graded as poor, intermediate or high, with the scores ≥6 were considered as high quality studies. Disagreements were resolved by discussions with a third investigator.

### Statistical analysis

Extracted data were pooled using the STATA software package (version 15.1; Stata Corporation, College Station, Texas USA). Continuous outcomes were presented as Weighted Mean Difference (WMD) and the 95% Confidence Interval (CI) was used to evaluate the precision and significance of that point of estimate. Forest plot was tested under the fixed or random effects models. If there was considerable heterogeneity across the combined studies, the random effects model was considered. Higgins I^2^ statistic was used to test for heterogeneity between the included studies.^13^ Generally, I^2^ < 25% can be interpreted as an indicator of mild heterogeneity; I^2^ value between 25% and 50%, to moderate heterogeneity while I^2^ > 50%, to significant heterogeneity. To rule out over representation of results from a single study in the meta-analysis, we performed sensitivity analysis by eliminating each study individually. Funnel plot and Egger’s test were used to assess the potential publication bias. All statistical tests were two-sided, and *p*-value of <0.05 were considered statistically significant.

## Results

### Characterization of the selected studies

**Figure 1:**
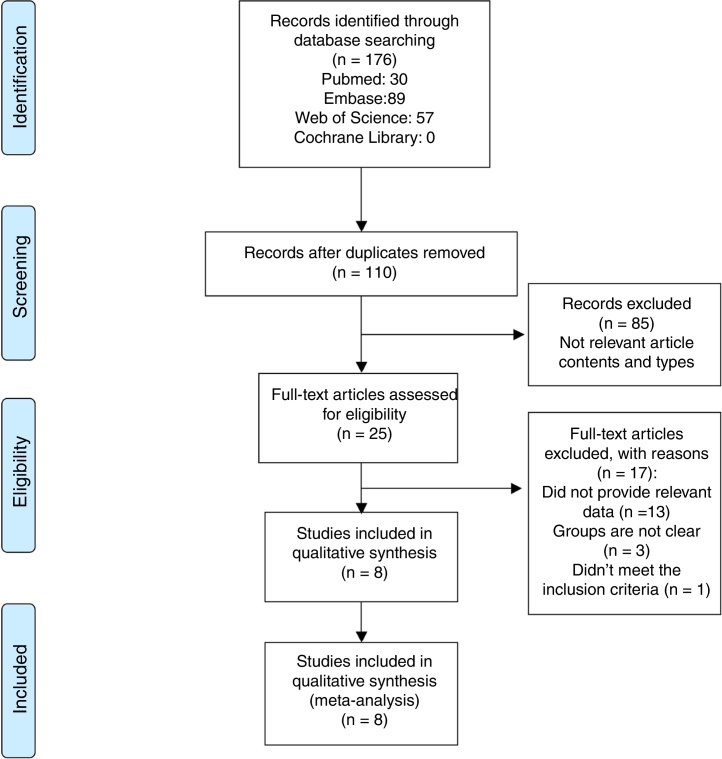
Flow diagram of the study selection process.

The flow diagram of this study was presented in the Fig. 1. Our research strategy identified 176 articles including 30 articles from Pubmed, 89 articles from Embase, 0 articles from Cochrane Library and 57 articles from Web of Science. One hundred and 10 studies were evaluated after removal of duplicates. After reviewing the tiles and abstracts, 25 studies were selected for full-text reading. Finally, eight studies were included in the current meta-analysis. Study characteristics are summarized in the Table 1.^7^, ^14^, ^15^, ^16^, ^17^, ^18^, ^19^, ^20^ These clinical studies included 337 CRS patients and 179 healthy controls. All studies were published in English. Five studies 7, 14, 15, 16, 20 compared both CRSwNP and CRSsNP with controls. Three studies^7^, ^18^, ^19^ were carried out as prospective researches and the rest were retrospective ones. The majority of the literatures used 20 ng/mL as the cutoff value of vitamin D deficiency. The results of qualitative assessment of the included studies were shown in Table 1. Overall, most of the studies included in this meta-analysis had high quality.

**Table 1:** Characteristics of all included studies.

Author	Year	Country	Type of study	Nº of participants	Vitamin D level (Mean ± SD) (ng/mL)	Cutoff value	Baseline characteristics between the groups	NOS score
Mulligan^14^	2011	USA	Retrospective	CRS: 29 Control: 14	CRS: 36.62 ± 12.99 Control: 51 ± 4	<20 ng/mL	No significant differences in gender, race, BMI.	7
Mulligan^15^	2012	USA	Retrospective	CRS: 22 Control: 14	CRS: 30.07 ± 6.96 Control: 38.4 ± 14	<20 ng/mL	NG	6
Mulligan^16^	2014	USA	Retrospective	CRS: 85 Control: 21	CRS: 29.31 ± 15.91 Control: 37.48 ± 20.89	<32 ng/mL	NG	6
Carroll^17^	2016	USA	Retrospective	CRS: 13 Control: 6	CRS: 34.5 ± 21.1 Control: 20.8 ± 9.7	<20 ng/mL	The average age was 52 years for control patients and 50 years for CRSwNP patients. 25% of control patients were male and 47% of CRSwNP patients were male.	6
Erdag^18^	2016	Turkey	Prospective	CRS: 46 Control: 40	CRS: 13.38 ± 14.08 Control: 10.57 ± 6.44	<20 ng/mL	No significant differences in age, gender.	7
Mostafa^7^	2016	Egypt	Prospective	CRS: 30 Control: 19	CRS: 38.6 ± 28.2 Control: 63.3 ± 17.3	NG	No significant differences in age, gender.	7
Shanaki^19^	2017	Iran	Prospective	CRS: 45 Control: 44	CRS: 16.62 ± 5.16 Control: 26.08 ± 14.16	<20 ng/mL	NG	6
Wang^20^	2018	China	Retrospective	CRS: 67 Control: 21	CRS: 42.2 ± 11.49 Control: 54.1 ± 17.1	<20 ng/mL	No significant differences in age, gender, BMI, smoke history, atopic status and asthma	7

95% CI, 95% confidence intervals; WMD, weighted mean difference; CRS, chronic rhinosinusitis; CRSwNP, chronic rhinosinusitis with nasal polyps; CRSsNP, chronic rhinosinusitis without nasal polyps; NOS, Newcastle-Ottawa quality assessment scale; NG, not given.

### Association between serum vitamin D level and CRS

**Figure 2:**
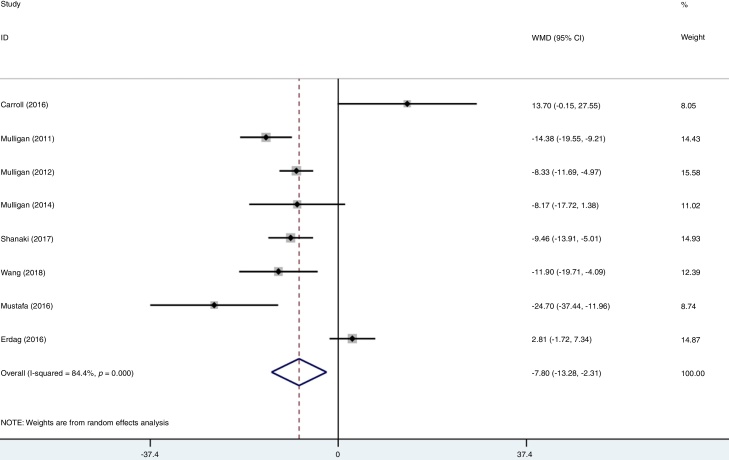
Forest plot of the association between serum vitamin D and participants.

A total of eight studies reported data for serum vitamin D among CRS and participants. Firstly, we combined the data of CRSwNP and CRSsNP into a single group of CRS according to Cochrane Handbook.^13^ Meanwhile, the individual data of CRSwNP or CRSsNP would be generally defined as CRS when the study just compared one of them with controls. After data consolidation, we made the first meta-analysis under the random effects model. Results showed that there was significant difference in the serum vitamin D in CRS patients when compared to the healthy controls (WMD = −7.80, 95% CI −13.28 to −2.31). In the first group meta-analysis, there was a remarkable heterogeneity (I^2^ = 84.4%, *p* = 0.000) (Fig. 2).

### Heterogeneity and subgroup analysis

**Figure 3:**
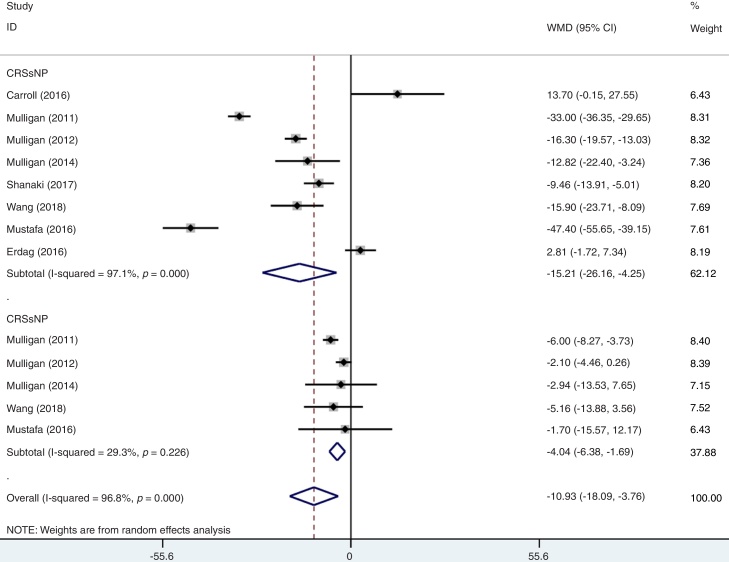
Forest plot of the association between serum vitamin D and CRS stratified by phenotypes of CRS.

**Figure 4:**
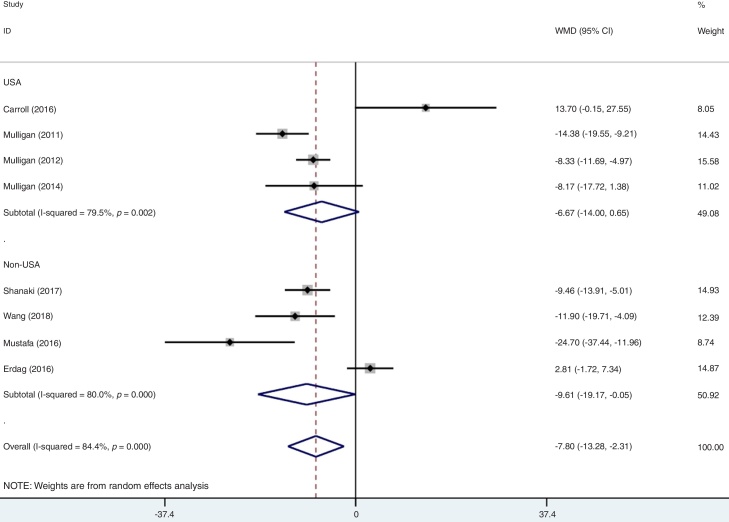
Forest plot of the association between serum vitamin D and CRS stratified by geographic difference.

**Figure 5:**
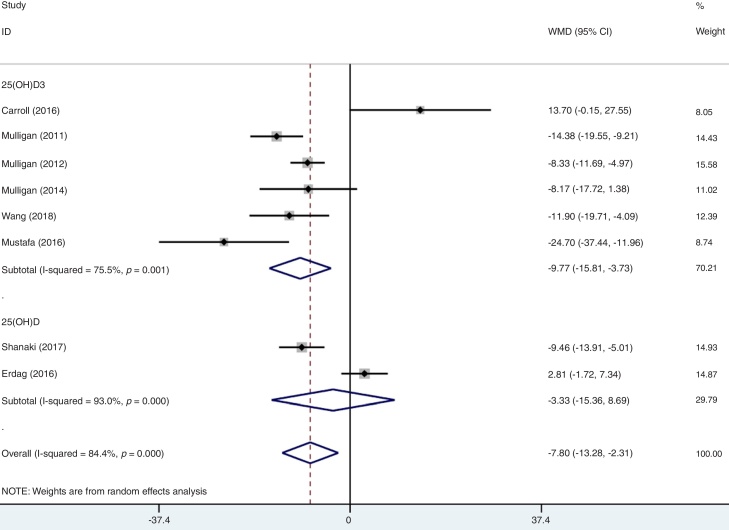
Forest plot of the association between serum vitamin D and CRS stratified by type of biomarker.

**Figure 6:**
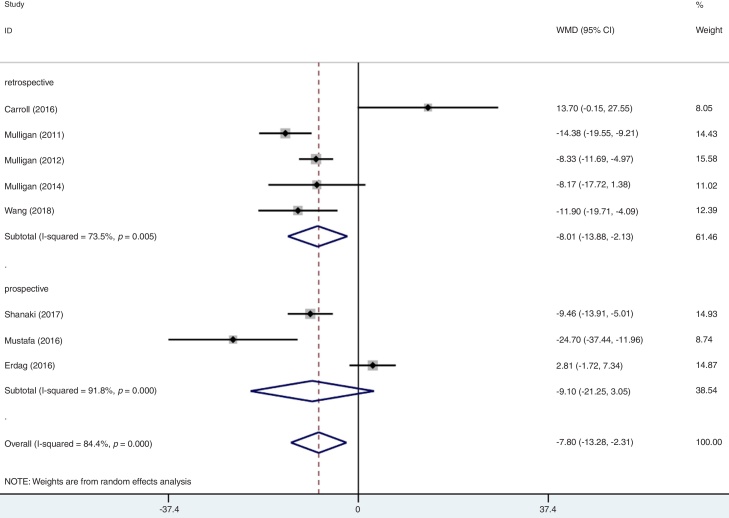
Forest plot of the association between serum vitamin D and CRS stratified by study design.

As mentioned above, moderate level of heterogeneity was detected in the analysis of the eight studies. Consequently, subgroup analyses were carried out according to the phenotypes of CRS (CRSwNP and CRSsNP), types of biomarker (25(OH)D3 and 25(OH)D), geographic area (USA and non-USA) and study design (Table 2). In an analysis stratified by phenotypes of CRS, the summary WMD from CRSwNP (WMD = −15.21; 95% CI −26.16 to −4.25, I^2^ = 97.1%, *p* = 0.000) showed that there was significant association for the lowest serum vitamin D with CRSwNP and CRSsNP subjects (WMD = −4.04; 95% CI −6.38 to −1.69, I^2^ = 29.3%, *p* = 0.226) (Fig. 3). When stratified by geographical region, studies conducted in USA and non-USA showed different results. There was no significant association between vitamin D and CRS among USA citizens (WMD = −6.67; 95% CI −14.00 to 0.65, I^2^ = 79.5%, *p* = 0.002), while lower vitamin D level was associated with CRS in other Countries (WMD = −9.61; 95% CI −19.17 to −0.05, I^2^ = 89.0%, *p* = 0.000) (Fig. 4). The subgroup analysis based on types of biomarker (25(OH)D3 and 25(OH)D) indicated that the mean levels of serum vitamin D were significantly lower between CRS patients and controls in studies that assessed the serum vitamin D status by measuring 25(OH)D3, but the same phenomenon was not found for the 25(OH)D assessed studies (WMD = −3.33; 95% CI −15.36 to 8.69, I^2^ = 93.0%, *p* = 0.000) (Fig. 5). The subgroup analysis of retrospective designed studies showed a significant association between lower serum vitamin D status and CRS (WMD = −8.01; 95% CI −13.88 to −2.13, *p* = 0.005) but with a high heterogeneity (I^2^ = 73.5%) and no such phenomenon was seen in prospective ones (WMD = −9.10; 95% CI −21.25 to 3.05, I^2^ = 91.8%, *p*  = 0.000) (Fig. 6).

**Table 2:** A summary of WMD for the overall and 95% CIs of vitamin D and CRS.

Subgroup	Nº of studies	Nº of patients	Nº of controls	WMD (95% CI)	*p*-value	Heterogeneity I^2^ (%)
Overall	8	337	179	−7.80 (−13.28 to −2.31)	0.000	84.4
Phenotype						
CRSwNP	8	220	179^a^	−15.21 (−26.16 to −4.25)	0.000	97.1
CRSsNP	5	117	89^a^	−4.04 (−6.38 to −1.69)	0.226	29.3
Biomarker						
25(OH)D3	6	246	95	−9.77 (−15.81 to −3.73)	0.001	75.5
25(OH)D	2	91	84	−3.33 (−15.36 to 8.69)	0.000	93.0
Geographic area						
USA	4	149	55	−6.67 (−14.00 to 0.65)	0.002	79.5
Non-USA	4	188	124	−9.61 (−19.17 to −0.05)	0.000	89.0
Study design						
Retrospective	5	216	76	−8.01 (−13.88 to −2.13)	0.005	73.5
Prospective	3	121	103	−9.10 (−21.25 to 3.05)	0.000	91.8

95% CI, 95% confidence intervals; WMD, weighted mean difference; CRSwNP, chronic rhinosinusitis with nasal polyps; CRSsNP, chronic rhinosinusitis without nasal polyps; 25(OH)D, 25-Hydroxyvitamin D.

a Most of the included studies compared both the CRSwNP and CRSsNP with controls, leading to the number of controls were repeatedly counted.

### Sensitivity analysis and publication bias

**Figure 7:**
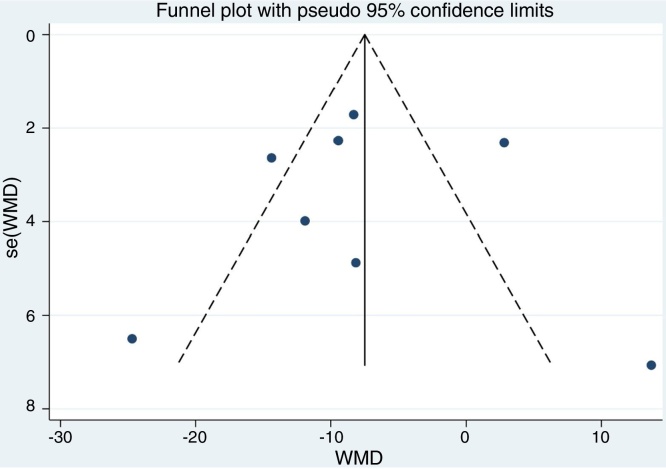
Funnel plot of meta-analysis of CRS vs. control studies.

**Figure 8:**
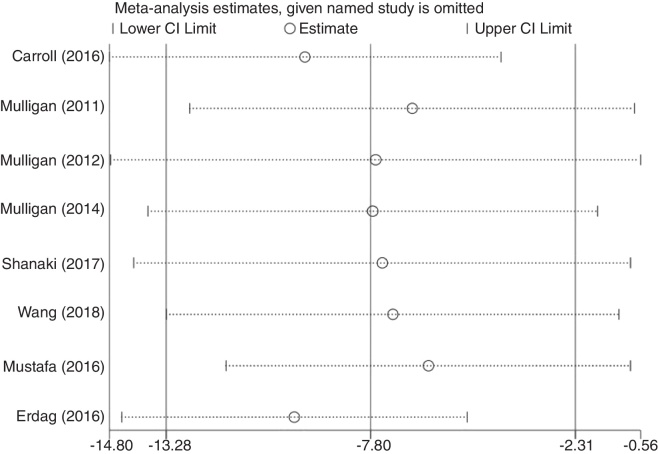
Sensitivity analysis on the relationship between serum vitamin D and CRS.

**Figure 9:**
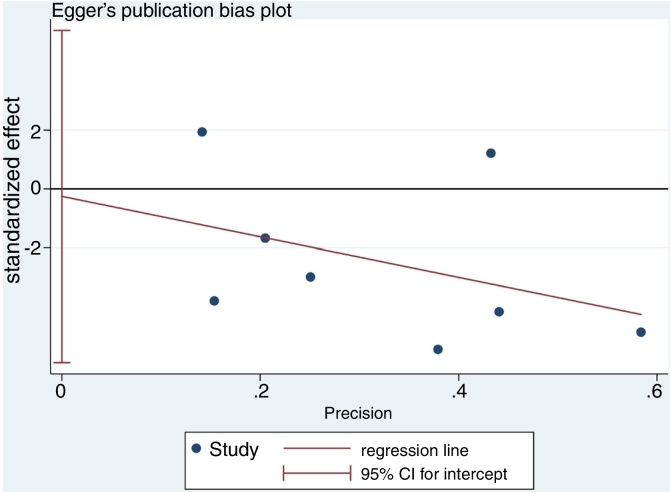
Egger’s test for publication bias.

Although the funnel plot manifested a slight asymmetrically for the association between low serum vitamin D level and CRS (Fig. 7). The results of sensitivity analysis suggest that the influence of each individual data set on the pooled WMDs was not significant (Fig. 8). *p*-value for the Egger’s test was 0.916, which suggested that there was no evidence of publication bias in this meta-analysis (Fig. 9).

## Discussion

Playing an essential role in anti-inflammation and anti-proliferation, vitamin D is known as an immunomodulator. After initial hydroxylation in liver, vitamin D transforms to the prohormone calcidiol (25(OH)D3), which would circulate and active to calcitriol (1.25(OH)D3) by 1-α-hydroxylase in peripheral tissues. After binding to the intracellular Vitamin D Receptor (VDR), a number of cell signaling pathways are activated.^21^, ^22^ In vitro studies have shown that 1.25(OH)D3 could reduce the expression of pro-inflammatory cytokines (IL-6, IL-8, RANTES, eotaxin) by human sinonasal epithelial cells.^23^ Furthermore, vitamin D is necessary for T-regulatory cell activity and T-cell responses to infection.^24^ Through these features mentioned above, vitamin D was considered to be one of the important factors that could influence the pathogenesis of CRS. However, contradictory conclusions were summarized by different research centers. Therefore, we carried out this first meta-analysis to assess the potential relationship between serum vitamin D level and CRS. The searching strategy was performed with no period limitation. Nevertheless, we found only eight studies eligible for our analysis.

A total of 516 cases in 8 relevant studies were identified in the current meta-analysis. The results indicated that the low serum vitamin D level was significantly associated with CRS under the random effect model. Low systemic vitamin D level might interfere with natural mechanisms to limit the mucosa inflammation, anti-proliferative and anti-angiogenic properties leading to episodes of CRS.^25^, ^26^, ^27^ Patients suffering from CRS presented a lower level of serum vitamin D than healthy subjects. This analysis result was in accord with a study by Faruk, which indicated that vitamin D is protective against the development of sinusitis and that the lack of vitamin D might induce the development of pre-septal cellulitis. Meanwhile the symptoms and signs of CRS could be relieved by administration of vitamin D.^28^

However, there was high heterogeneity in the strength of associations between studies. To further explore the relationship, stratified analyses were performed based to the two different phenotypes of CRS and the finding of the subgroup analysis suggested that lower serum vitamin D was related to both CRSwNP and CRSsNP patients, especially to the CRS with polyps. This result was in line with the retrospective studies, which showed that vitamin D deficiency status was more prevalent in CRSwNP subjects.^7^, ^8^, ^9^ Meanwhile, Wang and Mostafa found the 25(OH)D3 level in CRSwNP patients was significant lower than that in CRSsNP ones.^7^, ^29^ Studies have shown that vitamin D status is associated with systemic expression of dendritic cells, activation of T-cells and basic fibroblast growth factor in patients with CRSwNP. Moreover, it might play an anti-inflammatory function in CRSwNP, reducing the proliferation of nasal polyp fibroblasts and the secretion of matrix metalloproteinases and cytokines.^17^

Including sex, race, season, body mass index and geographic area, these factors were known to randomly influence the serum 25(OH)D. Pinto et al. found that serum levels in African American patients with CRS were significantly lower than those in race- and sex-matched control groups while there was no significant difference between white subjects when using the same matched strategy.^9^ It indicates that race or diet habit might be an important factor that could affect the serum vitamin D level. In this meta-analysis, we separate thestudies into two sub-groups (USA and Non-USA) to explore the heterogeneity of the included studies. As Fig. 4 demonstrated, Non-USA group seemed to present difference of serum vitamin D between CRS and healthy. The different results of the two groups might had interference by some other factors such as geographic latitude or patients’ age. For example, data have shown that people in northern latitude always present a low serum vitamin D level for leak of sun exposure. Moreover, aging has also been noted to affect serum vitamin D status.^30^ Homogeneity of the subjects should be noticed in future.

To our knowledge, this is the first cumulative analysis comparing serum vitamin D level of CRS with healthy ones. The strengths of the present study are: (1) Our analysis included all available studies that investigated the association between serum vitamin D status and CRS and the searching strategy and analysis were performed in an impartial and systematic manner in line with Cochrane standards. (2) The sensitivity analysis and publication bias detection suggested that the conclusion of this meta-analysis is quite stable. Although our results provide compelling evidence to support the phenomenon that serum vitamin D status might be a risk factor for CRS in the general population, limitations were also found in our study as other observational studies. For instance, there were only 8 studies included, and some of them were not of extreme high quality. The fact that no RCTs trials included might induce an inescapable bias. Most of the included studies were retrospective studies, which implied a case-control design, and therefore there was a selection and recall bias. Second, heterogeneity existed between different studies. Through the subgroups analysis, we noticed that the phenotype of CRS might be the reason of heterogeneity in this meta-analysis. Finally, we eliminate some studies published in non-English language, which might lead to a deficiency of data.

## Conclusions

In summary, our meta-analysis of 8 studies illustrates the lower serum vitamin D status in CRS patients, which indicates that people might get benefit from appropriate vitamin D supplementation. Therefore, due to the heterogeneity of the subjects, more well-designed prospective RCTs should be carried out to further validate these findings in for the general population in the future.

## Funding

This work was supported by a Grant of the Deep Underground Space Medical Center Research Foundation of Sichuan University (DUGM201804).

## Conflicts of interest

The authors declare no conflicts of interest.
